# Oseltamivir-induced behavioral changes in a female Lebanese adolescent: a case report of a usual drug with unusual side effect

**DOI:** 10.2144/fsoa-2019-0162

**Published:** 2020-06-26

**Authors:** Rabih Fares, Antoine Zgheib, Rabih Hallit, Souheil Hallit

**Affiliations:** 1 Faculty of Medicine & Medical Sciences, Holy Spirit University of Kaslik (USEK), Jounieh, Lebanon; 2 Department of Internal Medicine, Notre Dame Maritime Hospital, Jbeil, Lebanon; 3 Infectious Disease department, Notre Dame des Secours University Hospital, Byblos, Lebanon; 4 Infectious Disease department, Bellevue Medical Center, Mansourieh, Lebanon; 5 INSPECT-LB: Institut National de Sante Publique, Epidemiologie Clinique et Toxicologie – Liban, Beirut, Lebanon

**Keywords:** abnormal behavior, adolescent, antiviral drug, influenza, neuropsychiatric, oseltamivir

## Abstract

Neuropsychiatric symptoms, including abnormal behavior, after oseltamivir use among influenza patients have been of concern. In recent years, many case reports of neuropsychiatric events during or after oseltamivir treatment were reported; however, literature review revealed no such cases in Lebanon. Oseltamivir is the most widely prescribed medication against influenza and is generally well tolerated, causing random gastrointestinal disturbances, including nausea, vomiting, diarrhea and abdominal pain. However, in rare instances, it has been reported to stimulate behavioral activities in adolescents. We report herein a case of an oseltamivir-related neuropsychiatric event in a female adolescent in Lebanon, which resolved 2 days after stopping the drug.

Oseltamivir, a neuraminidase inhibitor, is the most prescribed drug for the treatment and prophylaxis of influenza in persons at high risk of complications worldwide [1,2]. In the Lebanese population, its use is high as well. Nevertheless, oseltamivir-induced neuropsychiatric distress has been reported, especially among the Japanese pediatric population [3], which pressed the Japanese Ministry of Health to restrict its use among children and adolescents aged 10–19 years [4,5]. However, studies are still inconclusive about whether a true association exists between oseltamivir use and neuropsychiatric warning signs [6–8]. A literature search revealed few cases of neuropsychiatric concerns following oseltamivir use [9–12]. We present a case of oseltamivir-induced abnormal behavior in a female Lebanese adolescent.

## Case

A previously healthy 16-year-old girl came to the emergency department after developing headache, fever, photophobia, neck stiffness and nausea. She reported having a good health status, with no use of tobacco, alcohol or illicit drugs, until 1 day before presentation when she began to have a mild sore throat, nasal congestion and temperature of 39°C associated with headache and myalgia.

On examination, her temperature was 38.5°C, blood pressure 115/68 mmHg, pulse 90 beats per min, respiratory rate 18 respirations/min and a 95% oxygen saturation. The patient appeared ill, with an intense photophobia, mild-to-moderate neck stiffness and an erythematous oropharynx. The remaining physical exam was normal. Chest radiography and computed tomography of the brain, without intravenous contrast dye were done, with results returning normal. Lumbar puncture was performed; results of the cerebrospinal fluid (CSF) analysis are shown below in Table 1.

**Table 1.** Laboratory data of the patient’s cerebrospinal fluid analysis.

Variable	Reference range	On admission	
Culture		No growth after 8 days of admission	
Serology:			
– CMV IgM	Negative <0.85	0.34	
– EBV IgM	Negative <0.5	0.06	

CMV: Cytomegalovirus; EBV: Epstein–Barr virus.

Intravenous ceftriaxone (2 g every 12 h) and vancomycin (loading dose of 30 mg/kg followed by 20 mg/kg every 12 h) and acetaminophen (1 g every 6 h) were administered empirically at admission following lumbar puncture and normal saline was infused. Cultures of urine and blood specimens were sterile after 1 and 8 days, respectively. Urinalysis was negative. The electrocardiogram was normal.

During the following hours, fever and chills resolved but headache persisted. A rapid test of a specimen from a nasopharyngeal secretions came back negative for influenza A and B antigens, but the influenza PCR was positive for flu A and H1N1 (Table 2). Therefore, antibiotics were stopped and oseltamivir was initiated orally on the same day following admission, with 1000 mg paracetamol given every 6 h as needed.

**Table 2.** Molecular biology of the nasal specimen.

PCR: influenza A/B (GeneXpert)	
Specimen	Nasal
Flu A (41 species)	POSITIVE
Flu B (12 species)	NEGATIVE
Flu A H1N1	POSITIVE

Two days after hospitalization, in an isolated ward, the patient started acting in an atypical behavior; she was fearful for no reason over getting out of bed, complaining in a shouting way of neck tenderness and headache and was abnormally frightened of being left alone. A second CT scan and neck x-ray yielded normal results. The patient seemed tense, with her agitation worsening at night. Benzodiazepines were started after consulting a psychiatrist. No deficit in attention or inappropriate speech were noted. Given this picture, the administration of oseltamivir was stopped on the 4th day. The patient was discharged home with a prescription of clonazepam 2 mg twice daily for 1 week post-discharge and acetaminophen every 6 h as needed. Forty-eight hours after discharge and after stopping oseltamivir, the patient’s mood and behavior were back to normal and the patient returned to her normal daily activities.

## Discussion

Teenager patients attending schools are highly susceptible to influenza infections and thus, are prone to be treated with oseltamivir that accelerates time to symptoms’ relief, reduces the risk of lower tract pulmonary complications and hospital admissions [13]. Yet, it has been reported in many countries that oseltamivir use can cause neuropsychiatric events, with more than 100 cases of abnormal behavior and 70 deaths reported in Japan alone [14].

Other reports claimed that influenza A H1N1 is associated with a variety of neurologic complications including seizures (febrile or afebrile), encephalitis, encephalopathy and Reye’s syndrome [15]. In addition, the A/H1N1 influenza virus can cause disorientation, confusion and irritability [13]. Some prospective clinical studies found no evidence of higher neuropsychiatric adverse events due to oseltamivir compared with placebo. Such examinations support the fact that the flu virus may be responsible of such adverse effects rather than the treatment itself [16]. However, many case reports strongly support a temporally association between oseltamivir use and neuropsychiatric events [17]. Since the A/H1N1 influenza virus and oseltamivir can cause neuropsychiatric manifestations, there might be an even higher risk in presenting them when both risk factors are present. The absence of previous personal or familial history of psychiatric illness supported this theory in our patient.

The oseltamivir-induced neuropsychiatric events can happen promptly, 24 h after its administration or after a longer delay. A randomized controlled trial conducted in 2014 showed that the odds of a patient suffering from a neuropsychiatric alteration after oseltamivir use increase by threefold compared with placebo [18]. A recent study showed that this risk is 1.90-fold higher in the 2-day time window, with the highest risk observed among patients aged 10–19 years [15]; these results corroborate those of a previous cohort study, which showed a 1.21-fold increase in the risk of neuropsychiatric adverse events and a 1.5-fold increase in the risk of getting those adverse events in patients aged 10–19 years in subjects treated with oseltamivir compared with not [8].

What made this case more suggestive is the temporal relationship between the onset of the neuropsychiatric symptoms that occurred 48 h after the initiation of oseltamivir and the quick symptoms’ withdrawal after stopping it, a relationship found in many previous case reports that favors the diagnosis of oseltamivir-induced neuropsychiatric symptoms. Oseltamivir-induced suicidal ideation has also been reported, especially in young adults [16]. The exact mechanism of oseltamivir-induced psychosis is unknown; however, a raise of dopamine levels in the medial prefrontal cortices of rats [19] and the agonistic activity of dopamine on D2 receptors through the inhibition of the neuraminidase (sialidase) that breaks down the sialic acid to glycolipids, have been incriminated in this process [14]. Another mechanism is the benzodiazepine-like central depression action triggered by oseltamivir (in its unchanged form) that causes abnormal behavior, delirium and hallucination [20].

Current clinical guidelines recommend early administration of antivirals to hospitalized patients with laboratory-confirmed or suspected influenza, where about 290,000–650,000 mortalities globally each year are linked to influenza [21]. In the coming years, further studies concerning the pharmacokinetics and pharmacodynamics of oseltamivir should be performed in subjects having inexplicable neuropsychiatric adverse events. This would allow providers to draw firm conclusions regarding the best dosage to use in children and adolescents, overcoming the behavioral disturbances that clinicians encounter in their practice. Such approach could help pharmaceutical companies improve their product and figure out which adverse events cause patient's dissatisfaction, since 82.9% of patients report lack of satisfaction with oseltamivir use when it comes to its neuropsychological side effects [22].

## Conclusion

To our knowledge, this is the first reported case of oseltamivir-associated unusual behavior in Lebanon. We hope that the present report will encourage Lebanese clinicians to monitor oseltamivir’s potential neuropsychiatric adverse events, as the extensive use of this drug will continue most likely for the prevention and treatment of influenza.

**Executive summary**

- Oseltamivir is licensed for the treatment of influenza and for the post-exposure and seasonal prophylaxis on influenza in adults and children aged 1 year or older.
- Oseltamivir is well tolerated with the most frequent reported adverse events being nausea and vomiting, which are usually mild and transient.
- Oseltamivir use has been associated with the development of transient neuropsychiatric events (i.e., self-injury or delirium) in a temporal relationship manner.

## References

[1] Centers for Disease Control and Prevention. Updated Interim Recommendations for the Use of Antiviral Medications in the Treatment and Prevention of Influenza for the 2009–2010 (2009). www.cdc.gov/h1n1flu/recommendations.htm

[2] Centers for Disease Control and Prevention. Influenza antiviral medications: summary for clinicians (2019). www.cdc.gov/flu/professionals/antivirals/summary-clinicians.htm#modalIdString_CDCTable_1

[3] Fuyuno I Tamiflu side effects come under scrutiny. Nature 446(7134), 358–359 (2007). 10.1038/446358a 17377552

[4] Hama R Oseltamivir's adverse reactions: fifty sudden deaths may be related to central suppression. BMJ 335(7610), 59 (2007). 17626921 10.1136/bmj.39262.448877.BE PMC1914480

[5] Maxwell SR Tamiflu and neuropsychiatric disturbance in adolescents. BMJ 334(7606), 1232–1233 (2007). 17569896 10.1136/bmj.39240.497025.80 PMC1892497

[6] Harrington R, Adimadhyam S, Lee TA, Schumock GT, Antoon JW The relationship between oseltamivir and suicide in pediatric patients. Ann. Fam. Med. 16(2), 145–148 (2018). 29531106 10.1370/afm.2183 PMC5847353

[7] Yorifuji T, Suzuki E, Tsuda T Oseltamivir and abnormal behaviors: true or not? Epidemiology 20(4), 619–621 (2009). 19367165 10.1097/EDE.0b013e3181a3d3f6

[8] Greene SK, Li L, Shay DK Risk of adverse events following oseltamivir treatment in influenza outpatients, Vaccine Safety Datalink Project, 2007–2010. Pharmacoepidemiol. Drug Saf. 22(4), 335–344 (2013). 23129321 10.1002/pds.3363

[9] Chung S, Joung YS Oseltamivir (tamiflu) induced depressive episode in a female adolescent. Psychiatry Investig. 7(4), 302–304 (2010). 10.4306/pi.2010.7.4.302 PMC3022319 21253416

[10] Ho LN, Chung JP, Choy KL Oseltamivir-induced mania in a patient with H1N1. Am. J. Psychiatry 167(3), 350 (2010). 10.1176/appi.ajp.2009.09101421 20194491

[11] Teng J, Lee T-S P01-403-Persistent mania and psychosis in a case of novel influenza a (H1N1) virus encephalitis. Eur. Psychiatry 26, 406 (2011).

[12] Perera S, Wellaketiya R Ostelamivir (Tamiflu) induced mania-a case report. Sri Lanka J.Psychiatry 9(1), 29–31 (2018).

[13] Dobson J, Whitley RJ, Pocock S, Monto AS Oseltamivir treatment for influenza in adults: a meta-analysis of randomised controlled trials. Lancet 385(9979), 1729–1737 (2015). 25640810 10.1016/S0140-6736(14)62449-1

[14] Shaik AB, Prabhu M, Shenoy S, Thomson SR Oseltamivir-induced neuropsychiatric symptoms. J. Pharmacol. Pharmacother. 9(1), 43 (2018).

[15] Kang H-R, Lee E-K, Kim WJ, Shin J-Y Risk of neuropsychiatric adverse events associated with the use of oseltamivir: a nationwide population-based case-crossover study. J. Antimicrob. Chemother. 74(2), 453–461 (2019). 30418537 10.1093/jac/dky445

[16] Toovey S, Rayner C, Prinssen E Assessment of neuropsychiatric adverse events in influenza patients treated with oseltamivir: a comprehensive review. Drug Saf. 31(12), 1097–1114 (2008). 19026027 10.2165/0002018-200831120-00006

[17] Jones M, Tett SE, Del Mar C Psychiatric adverse events in oseltamivir prophylaxis trials: novel comparative analysis using data obtained from clinical study reports. Pharmacoepidemiol. Drug Saf. 27(11), 1217–1222 (2018). 30209862 10.1002/pds.4651

[18] Jefferson T, Jones MA, Doshi P Neuraminidase inhibitors for preventing and treating influenza in healthy adults and children. Cochrane Database Syst. Rev. , CD008965 (2014) (Epub ahead of print). 10.1002/14651858.CD001265.pub3 PMC10941719 20166059

[19] Lan C-C, Liu C-C, Chen Y-S Acute exacerbation of psychiatric symptoms during influenza treatment with oseltamivir in chronic schizophrenia. J. Chin. Med. Assoc. 78(6), 374–376 (2015). 25823677 10.1016/j.jcma.2014.05.017

[20] Alsop DC, Fearing MA, Johnson K, Sperling R, Fong TG, Inouye SK The role of neuroimaging in elucidating delirium pathophysiology. J. Gerontol. A Biol. Sci. Med. Sci. 61(12), 1287–1293 (2006). 17234822 10.1093/gerona/61.12.1287

[21] WHO. Up to 650 000 people die of respiratory diseases linked to seasonal flu each year (2017). www.who.int/en/news-room/detail/14-12-2017-up-to-650-000-people-die-of-respiratory-diseases-linked-to-seasonal-flu-each-year

[22] Antipov EA, Pokryshevskaya EB The effects of adverse drug reactions on patients’ satisfaction: evidence from publicly available data on tamiflu (oseltamivir). Int. J. Med. Inform. 125, 30–36 (2019). 30914178 10.1016/j.ijmedinf.2019.02.005

